# Advanced deep learning methods for molecular property prediction

**DOI:** 10.1002/qub2.23

**Published:** 2023-11-20

**Authors:** Chao Pang, Henry H. Y. Tong, Leyi Wei

**Affiliations:** ^1^ School of Software Shandong University Jinan China; ^2^ Joint SDU‐NTU Centre for Artificial Intelligence Research (C‐FAIR) Shandong University Jinan China; ^3^ Centre for Artificial Intelligence Driven Drug Discovery Faculty of Applied Science Macao Polytechnic University Macao China

**Keywords:** dataset, deep learning, molecular property prediction, molecular representations

## Abstract

The prediction of molecular properties is a crucial task in the field of drug discovery. Computational methods that can accurately predict molecular properties can significantly accelerate the drug discovery process and reduce the cost of drug discovery. In recent years, iterative updates in computing hardware and the rise of deep learning have created a new and effective path for molecular property prediction. Deep learning methods can leverage the vast amount of data accumulated over the years in drug discovery and do not require complex feature engineering. In this review, we summarize molecular representations and commonly used datasets in molecular property prediction models and present advanced deep learning methods for molecular property prediction, including state‐of‐the‐art deep learning networks such as graph neural networks and Transformer‐based models, as well as state‐of‐the‐art deep learning strategies such as 3D pre‐train, contrastive learning, multi‐task learning, transfer learning, and meta‐learning. We also point out some critical issues such as lack of datasets, low information utilization, and lack of specificity for diseases.

## 1 INTRODUCTION

*De novo* drug design is an expensive and time‐consuming process with a high failure rate, which takes more than 10 years and costs around 2.6 billion US dollars for a new drug [1]. In addition, the success rate of launching a drug from Phase I clinical trial to market is less than 10% [2]. Therefore, how to accurately and efficiently predict molecular properties for the fast screening of drug candidates is a vital issue in drug discovery. In order to solve this problem, pharmacists introduced computer‐aided drug discovery (CADD) methods to accelerate the drug discovery process, such as virtual screening, pharmacophore modeling and quantitative structure–property relationship (QSPR) models. However, most of these methods require manual feature extraction and suffer from low prediction accuracy, which cannot meet the requirements.

Recently, artificial intelligence (AI) technologies, represented by deep learning, have achieved great success in biology and chemistry fields [3, 4, 5], which indicates the great potential and outstanding creativity of AI. Compared to traditional drug property prediction methods, deep learning models can eliminate the complicated feature engineering process, accelerate computation process, and increase availability of large data sets. Therefore, more and more deep learning models were proposed to predict molecular properties.

In this review, we focus on several crucial components of molecular property predictive models: molecular representations, commonly used datasets, and advanced deep learning methods. Among all molecular property predictive methods, we have selected a few of the most representative categories of them, including graph neural network (GNN)‐based methods, Transformer‐based methods, 3D pre‐training methods, contrast learning methods, multi‐task learning methods, transfer learning methods, and meta‐learning methods. Finally, we discuss the current challenges existing in molecular property prediction and future directions.

## 2 REPRESENTATION AND DATASETS

### 2.1 Molecular representations

A molecular representation is a representation of a molecule that can be read and processed by computers. In order to use deep learning methods and acquire accurate molecular property prediction results, it is necessary to find an appropriate molecular representation. Many molecular representations have been proposed to represent molecules and they can be divided into two types: fixed representations and learned representations. Fixed representations are obtained by processing the molecule using a fixed set of rules, including molecular fingerprints [6], simplified molecular input line entry system (SMILES) [7], international chemical identifier (InChI) [8], molecular graphs [9], etc. Learned representations are learned molecular embeddings obtained by GNN or other models. Since learned representations are not directly obtainable from molecules and cannot be understood by humans, we only introduce the most commonly used fixed representations in this section.

#### 2.1.1 SMILES

**FIGURE 1:**
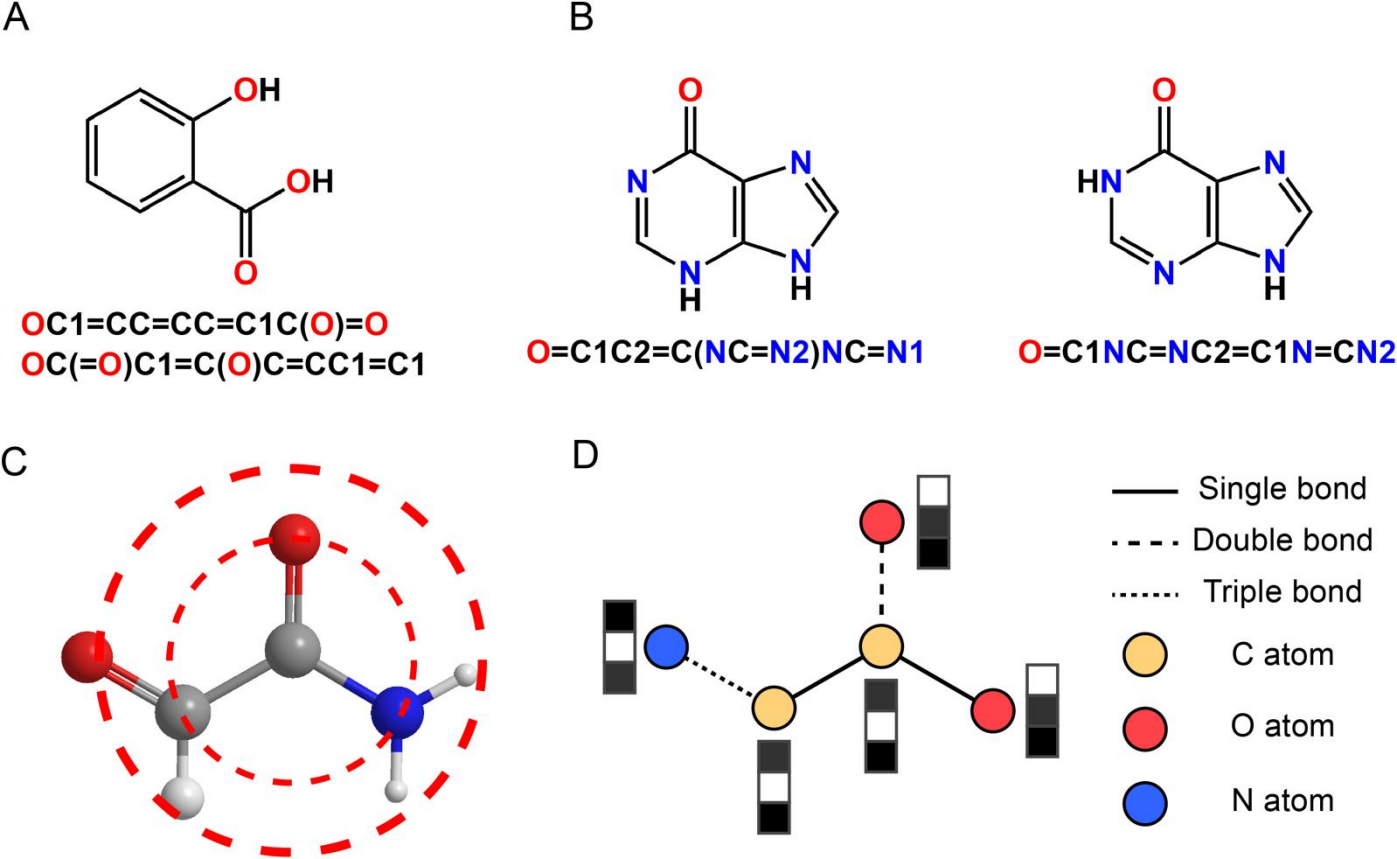
Commonly used molecular representations. (A) The same molecule can be represented as two different SMILES. (B) For two very similar molecules, their SMILES may look completely dissimilar. (C) Illustration of a molecular fingerprint. (D) Illustration of molecular graph. Each node and each edge in the molecular graph is labeled with its information.

SMILES describes a molecule in the form of a line notation using short ASCII strings. It represents atoms with one or two letters and single, double, and triple bonds with −, =, and #, respectively. Single bonds can be implicit to simplify representations. As a widely used molecular representation, SMILES has several prominent advantages as follows: First, it is a compact data format. A complex molecule can be represented by a few characters. Second, it is interpretable for humans and can be saved in text format very easily. Third, it is a text‐like representation, so methods from natural language processing (NLP) can be easily extended to handle SMILES [10]. However, SMILES also has some limitations: It is not a unique representation. It is possible for the same molecule to have several different SMILES. This drawback can be avoided by using canonical SMILES or data augmentation [11]. Next, SMILES cannot explicitly represent the similarity between molecules. Two similar molecules may be encoded into two obviously different SMILES. Figure 1A,B graphically illustrates these two drawbacks. Finally, SMILES needs to comply with strict syntactic restrictions (e.g., the parentheses in SMILES must appear in pairs and be closed), which leads to the possibility that the SMILES generated by the model is invalid. So, to address this drawback, Krenn et al. proposed SELF‐referencing embedded string (SELFIES), a 100% robust string‐based molecular representation [12]. It guarantees that all molecules generated by the model are valid.

#### 2.1.2 Molecular fingerprint

Molecular fingerprints are designed to represent the presence of substructures in molecules. It is usually noted as a sparse vector [13]. Among these molecular fingerprints, the extended connectivity fingerprint (ECFP) [6] is one of the most widely used molecular fingerprints. ECFPs are circular fingerprints that can be easy and fast to calculate and can efficiently represent different molecules in a huge chemical space. As shown in Figure 1C, the atoms/bonds are expected to increase as a circle grows around one atom in each iteration. Since ECFP features indicate the presence or absence of specific substructures in the molecule, the analysis results are interpretable for people. Because they can represent the substructures of molecules, they are well‐suited for tasks related to molecular property prediction. The final output of ECFPs is a hashed 32‐bit integer, which is very suited to be used as a feature of deep learning models due to its format.

#### 2.1.3 Molecular graph

A molecule can be represented quite naturally as an undirected graph G=(V,E), where nodes $\left\{v_{i}\in V\right.|\;i=1,2,3,\text{…},n\}$ represent atoms, edges $\left\{\left(v_{i},v_{j}\right)\in E\right.|\;i,j=1,2,3,\text{…},n\}$ represent bonds, and *n* denotes the number of atoms in the molecule. Nodes and edges in a molecular graph can be labeled to more accurately describe the various properties of atoms and bonds in the molecule. An example of molecular graphs is shown in Figure 1D. Furthermore, nodes in molecular graphs can also be used to represent substructures of molecules [14], to simplify the representation of complex molecules such as polymers, or to describe higher‐order structures of molecules. With the wide use of hypergraphs, some researchers have also used hypergraphs to represent molecules and obtained good results [15]. But graph representation also has some inevitable drawbacks. For instance, until now there is no good algorithm for determining if two graphs are isomorphic [16], that is, the graph isomorphism problem.

### 2.2 Datasets

In order to accurately evaluate the performance of molecular property prediction models, high‐quality data sets are essential. With the development of computational chemistry methods and the accumulation of data from wet experiments, the number of public datasets with information on molecular structure and molecular properties is constantly increasing. In this section, we present several categories of high‐quality datasets commonly used in molecular property prediction, including bioactivity, toxicity, and quantum mechanics. In addition, information on category, name, data size, and applicable prediction task type for each dataset is summarized in Table 1.

**TABLE 1:** Commonly used datasets in molecular property prediction.

Category	Dataset name	Prediction task	Data size	Ref.
Bioactivity	BACE	Classification	1513	[17]
	MUV	Classification	93,087	[18]
	HIV	Classification	41,127	[19]
	BBBP	Classification	1970	[20]
	NCI1	Classification	4110	[21]
Toxicity	SIDER	Classification	1427	[22]
	Tox21	Classification	7831	[23]
	ToxCast	Classification	8575	[24]
	ClinTox	Classification	1497	[25]
Quantum mechanics	QM7/QM7b	Regression	7160/7210	[26]
	QM8	Regression	21,786	[27]
	QM9	Regression	133,885	[28]

#### 2.2.1 Bioactivity

(1) BACE: The BACE dataset includes 1547 synthetic human β‐secretase 1 (BACE‐1) inhibitors reported in the scientific literature in the last few decades with quantitative (IC50) and qualitative (binary label) binding results [17]. (2) MUV: The Maximum Unbiased Validation (MUV) group is a benchmark dataset. Compounds in MUV are selected from PubChem by using a refined nearest neighbor analysis. MUV was created with the aim of minimizing the impact of benchmark dataset bias on validation results and providing a tool for computer‐aided screening methods, such as virtual screening [18]. (3) HIV: The HIV dataset contains over forty thousand compounds and their ability information of inhibiting HIV replication, which was introduced by the Drug Therapeutics Program (DTP) AIDS Antiviral Screen [19]. The compounds in HIV are divided into three categories based on their ability to inhibit the HIV replication process: confirmed inactive (CI), confirmed active (CA), and confirmed moderately active (CM). (4) BBBP: The blood–brain barrier penetration (BBBP) dataset is a set of 1970 molecules that shows an ability to permeate the human blood–brain barrier [20], which is a very important barrier in the human body that protects the central nervous system by the strict screening of chemicals which are allowed to pass [29]. (5) NCI1: The NCI1 is a dataset established by the National Cancer Institute (NCI). It is a subset of a balanced dataset with 37 discrete labels in which chemical compounds are screened for their ability to inhibit the growth of a panel of human tumor cell lines [21].

#### 2.2.2 Toxicity

(1) SIDER: The Side Effect Resource (SIDER) is a database of drugs on the market and adverse drug reactions (ADR). Its current release, SIDER 4 dataset, collects 5880 ADRs, 1430 drugs, and 140,064 drug–ADR pairs [22]. (2) Tox21: The Tox21 database is a publicly available database that was created by “The Toxicology in the 21st Century (Tox21) program”. The data in Tox21 database was generated from stress pathway assays run and nuclear receptor signaling against Tox21 program’s 10,000‐compound library [23]. Tox21 database was created to help researchers develop better methods for toxicity assessment. (3) ToxCast: The ToxCast is a database created by “The US Environmental Protection Agency’s (EPA) ToxCast program” [24]. It contains over 3800 compounds screened by using in vitro high‐throughput screening (HTS) methods and is used to support the development of accurate and quick toxicity prediction models. (4) ClinTox: The ClinTox dataset includes 1497 drug molecules with their chemical structures and two labels: clinical trial toxicity and FDA approval status [25].

#### 2.2.3 Quantum mechanics

(1) QM7/QM7b: The QM7 dataset is a subset of GDB‐13, which is a database consisting of almost one billion synthetically accessible and stable organic molecules. The QM7 is composed of 7165 molecules containing up to 23 atoms (including seven heavy atoms such as C, N, O, and S) [26]. The QM7b dataset is an extension of the QM7 dataset for multi‐task learning [30]. (2) QM8: The QM8 dataset consists of over 20,000 small organic molecules and their properties predicted by machine learning models. All these molecules consist of up to 8 heavy atoms (C, N, O, and F) and are chemically synthesizable [27]. These machine learning models were trained on deviations of reference second‐order approximate coupled‐cluster singles and doubles spectra from time‐dependent density functional theory counterparts. (3) QM9: The QM9 dataset is composed of 134,000 stable small organic molecules of CHONF and their computed geometrical, energetic, electronic, and thermodynamic properties [28]. These molecules belong to a subset of 166 billion organic molecules within the GDB‐17 chemical universe and all 133,885 molecules in QM9 with up to 9 heavy atoms (C, N, O, and F).

## 3 DEEP LEARNING METHODS

With the wide application of AI technologies, researchers have proposed various deep learning methods for molecular property prediction. In this section, we focus on several classes of advanced deep learning methods and discuss the most representative approaches in each class.

### 3.1 GNN‐based methods

GNN is a framework for learning directly from graph‐structured data using deep learning, which can transform graph‐structured data into a canonical and standard representation and feed it into many different neural networks for training, achieving excellent results on tasks such as node classification, edge information propagation, and graph clustering. As early as 2013, Lusci et al. realized the importance of deep learning methods and showed how recursive neural networks (RNN) could be applied to molecular property prediction tasks [31]. In 2015, Duvenaud et al. introduced a convolutional neural network (CNN) operating directly on graphs for molecular property prediction [32]. They used molecular fingerprints as input to obtain molecular features and demonstrated that these data‐driven features learned by models are more interpretable to machines and have better performance on predictive tasks. However, molecular fingerprint is not a good representation of the structural information of a molecule. Subsequently, Coley et al. constructed feature vectors of atoms using atom and bond attributes in molecules and considered local chemical environment information within different neighborhood radii [33]. By directly inputting the complete molecular graph into CNN, the model learns to recognize atom cluster features, significantly improving the performance of the CNN model. Gilmer et al. reformulated existing models as message passing neural networks (MPNN) and leveraged MPNN to demonstrate state‐of‐the‐art results on quantum mechanical property prediction tasks for small organic molecules [34]. For using the geometric structure information in molecules more effectively, Wang et al. used graph structures with convolutional networks to discover the relationship of each atom and designed a convolution spatial graph embedding layer (C‐SGEL) to make full use of the spatial connectivity information of molecules [35]. They constructed a composite model by stacking multiple C‐SGEL and combining them with molecular fingerprints, and achieved the best results on several datasets.

### 3.2 Transformer‐based methods

Thanks to the powerful self‐attention mechanism it uses, the Transformer has achieved great success in NLP [36], but is not ahead of GNN for graph‐level prediction tasks. To address this problem, Ying et al. believed that the key to exploit the Transformer on graphs is that the structural information of graphs must be efficiently encoded into the model [37]. They proposed Graphormer and some simple but effective methods for encoding structural information to assist Graphormer to encode graph‐structured data better. However, labeled data for training models are very scarce, which hinders further development of molecular property prediction models. To address problems of scarcity of labeled data and poor generalization capability, Li et al. used large‐scale unlabeled molecules to pre‐train MPG, a GNN model combined with the Transformer [38]. But both pre‐training and format conversion from SMILES to graph require additional resources. Therefore, Zhu et al. proposed ST‐KD, an end‐to‐end SMILES Transformer boosted by knowledge distillation for molecular representation learning [39]. By transferring knowledge from graph Transformers to ST‐KD, ST‐KD achieves competitive results while consuming fewer resources. Although the Transformer avoids some limitations of GNN by encoding only the positions, Chen et al. show that this does not necessarily capture the similarity between nodes. They proposed a Structure‐Aware Transformer which extracts a subgraph representation located at each node to integrate the structural information into the original self‐attention before computing the attention [40]. In addition, to alleviate the problem of limited model capacity and ill‐defined pre‐training tasks, Li et al. introduced Knowledge‐guided Pre‐training of Graph Transformer (KPGT), which uses a high‐capacity model to model molecular graphs with their structural information and a pre‐training strategy with knowledge guidance to capture the enriched structural information and semantic knowledge in unlabeled molecular graphs [41].

### 3.3 3D pre‐train methods

Molecular graphs are usually modeled by molecular 2D topology, but molecular 3D geometry plays a more important role in predicting molecular properties. Therefore, some methods have been proposed to address this challenge. Liu et al. proposed the Graph Multi‐View Pre‐training (GraphMVP), which performs self‐supervised learning using the correspondence and consistency of the multi‐view information of molecules (2D topology and 3D geometry of molecules) [42]. It is the first pre‐trained molecular representation learning model that introduces 3D geometric information of molecules to enhance the 2D molecular graph representation learning process. Gao et al. proposed that neural network models can be pre‐trained using 3D geometry and atomic charge as inputs and molecular energy as labels [43]. They further proposed a force regularization technique to make the pre‐trained model more suitable for force field prediction tasks. Experimental results demonstrated that the learned representations contain adequate information about molecular structures. Jiao et al. proposed an equivariant energy‐based model for 3D pre‐training, which is invariant to rotation and translation [44]. Then they developed a node‐level pre‐training loss for force prediction and a graph‐level noise scale prediction task for further increasing the performance. Fang et al. proposed a novel geometry‐enhanced molecular representation learning method (GEM) [45]. It contains a specially designed geometry‐based GNN for simultaneous modeling of the atom, bond, and bond angles effect and several specialized self‐supervised learning strategies to learn molecular 3D geometry information at geometry‐level.

### 3.4 Contrastive learning methods

**FIGURE 2:**
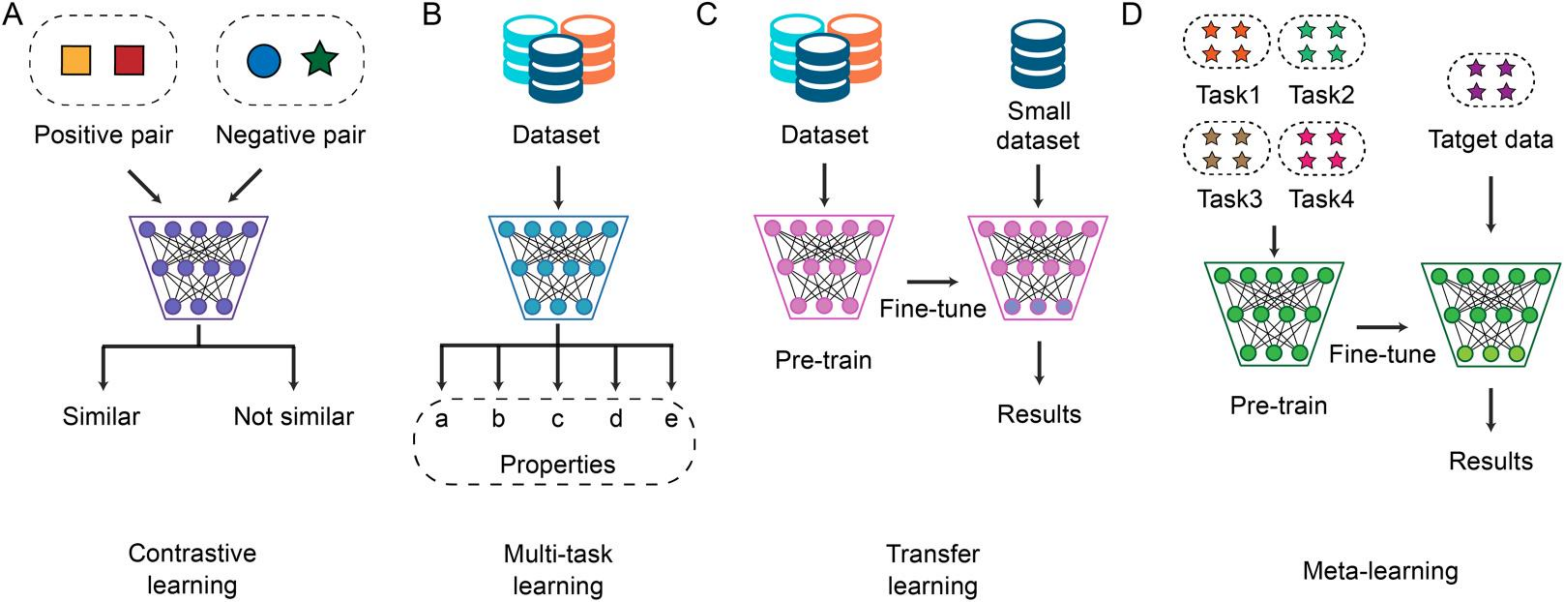
Four types of advanced deep learning methods. (A) Contrastive learning. The data will be divided into positive pairs and negative pairs. Contrast learning methods make the difference between positive pairs decrease and the difference between negative pairs increase. (B) Multi‐task learning. Multi‐task learning methods optimize multiple properties of molecules simultaneously. (C) Transfer learning. Transfer learning methods are pre‐trained on a larger dataset and then fine‐tuned on a smaller dataset. (D) Meta‐learning. Meta‐learning methods learn about the task itself, hoping that the model learns the ability to adjust hyperparameters.

Due to the expense and scarcity of labeled data, extending supervised learning methods to the huge chemical space (estimated to be about 10^60^) [46] is a great challenge. Contrast learning is a promising paradigm because it can effectively use unlabeled data for self‐supervised learning without human annotations. As shown in Figure 2A, contrast learning learns the information contained in unlabeled data by minimizing the difference between positive pairs and maximizing the difference between negative pairs. Wang et al. presented a molecular contrastive learning method for molecular representation learning via GNN called MolCLR, a self‐supervised learning framework using ∼10 million unlabeled molecular data [47]. They proposed three data augmentation methods for molecular graphs, including subgraph removal, atom masking, and bond deletion, and used a contrastive estimator to maximize the consistency of increments from the same molecule while minimizing the consistency of different molecules. Experiments demonstrate that the MolCLR pre‐trained GNN achieves significant improvements in multiple benchmarks and exhibits better generalization compared to supervised methods. Fang et al. further integrated domain knowledge into the semantics of graphs based on previous work and proposed knowledge‐enhanced contrastive learning (KCL) framework [48]. KCL summarizes microscopic associations between elements and the associations between atoms that share common properties but are not directly connected by bonds. Similarly, Sun et al. also noticed the importance of local domain knowledge and proposed MoCL, which leverages both local‐level and global‐level domain knowledge [49]. In terms of local‐level domain knowledge, they proposed a new augmentation scheme in which a substructure in the molecule is substituted by a bioisostere to introduce a portion of change without drastically altering properties of the molecule. In addition, Yin et al. made improvements in domain knowledge too. They proposed AutoGCL, which uses a set of learnable graph view generators coordinated by automatic enhancement policies [50]. The graph view generators learn a probability distribution of input graphs, thus preserving the most representative structure of the original graph while introducing sufficiently enhanced variances throughout the comparison learning process when generating each comparison sample.

Recently, some researchers noticed the importance of molecular geometry information in comparative learning methods, and they started to combine both of them to better predict molecular properties. Li et al. proposed a comparative learning method using both 2D and 3D views of the molecule called GeomGCL [51]. Specifically, they designed a multi‐view geometric message passing network to simultaneously exploit the 2D topology and 3D geometry of the molecule and a geometric graph comparison strategy that allows 2D topology views and 3D geometry views to collaborate and monitor each other. However, although 3D geometry can enhance the model performance, it is difficult to obtain in many cases. Stärk et al. used a 3D infomax method to maximize the information consistency between learned 3D geometry vectors and GNN representations [52]. Then, in the fine‐tuning phase, GNN can still generate implicit geometric information when only 2D information of the molecule is provided as input.

### 3.5 Multi‐task learning methods

In contrast to single‐task models that optimize for one property at a time, multi‐task learning methods optimize for multiple properties simultaneously (Figure 2B). On multiple GNNs, multi‐task learning models often outperform single‐task models using the same architecture. This conclusion has been confirmed by the study of Capela et al. [53]. They also found that the performance improvement is more pronounced on smaller datasets and multi‐task learning models have a wider distribution of GNN weights during training. Zubatyuk et al. integrated multi‐task learning with chemical knowledge and presented AIMNet, a chemically inspired and modular deep neural network [54]. They used this neural network to learn multiple patterns of atomic states in molecules, and the resulting model showed competitive results on multiple benchmark datasets. AIMNet trains multiple molecular properties simultaneously with multimodal and multi‐task training techniques to reduce the computational complexity of prediction. However, the amount of labeled data per task in multi‐task learning is too limited and needs to be supplemented with additional data. Liu et al. systematically studied the relation graph between different tasks in multi‐task learning [55]. They further proposed to find and model the relationship between different prediction tasks in latent space and output space. These representations effectively capture the similarity of tasks and can be further used for the prediction of different tasks.

### 3.6 Transfer learning methods

To alleviate the problem of scarce data in partial property prediction tasks, some researchers introduced a transfer learning strategy into molecular property prediction. Transfer learning methods are first trained on a large dataset and then fine‐tuned for a portion of trained network (usually the last layer) on a small target dataset, as shown in Figure 2C. Lentelink et al. thought that molecular images contain enough information that can be used for predicting molecular properties because experienced chemists can derive the properties of molecules by analyzing their structures [56]. They constructed a deep CNN model by migration learning and pre‐trained the model using images of molecules from the GDB‐17 dataset [57]. The obtained model was fine‐tuned on the ESOL dataset [58] and used for solubility prediction. Zhong et al. also combined molecular images with CNN to establish QSARs. For transfer learning, they used a DenseNet121 [59] that was pre‐trained on the ImageNet [60] and fine‐tuned it to predict compound rate constants toward OH radicals. Results show that transfer learning can significantly improve the predictive performance and robustness of the models. For some very small datasets, transfer learning can also work very well. Chen et al. proposed MRlogP, which first learns on a large number of low‐precision predicted log values and then is turned on a small, accurate dataset of 244 drug‐like molecules [61]. Their work offered the possibility of using limited high‐precision experimental measurements for accurate physicochemical property prediction. Li et al. took another perspective and used the similarity between molecular attribute prediction tasks to alleviate the data scarcity problem [62]. They proposed MoTSE to accurately estimate the task similarity and capture the inherent relation between molecular properties. Test results demonstrate that the obtained task similarity can effectively increase the performance of transfer learning in predicting molecular properties.

### 3.7 Meta‐learning methods

Similarly, to solve the problem of sparsely labeled data, meta‐learning methods have been used by researchers. Meta‐learning, that is, learning to learn, aims to learn how to adjust the hyperparameters of the model. Unlike other models that learn the data in the task, meta‐learning learns the task itself, which is illustrated in Figure 2D. In recent years, advances in meta‐learning made it possible to reach high performance in few‐shot learning. Nguyen et al. evaluated the transferability of GNN initializations that are learned through the model‐agnostic meta‐learning algorithm on molecular property prediction tasks [63]. They simulated the low resource case on the CHEMBL20 dataset [64] and the experimental results showed performance of meta‐initializations is comparable to or better than multitask pre‐training baselines on the majority of tasks including all out‐of‐distribution tasks and 16 of the 20 in‐distribution tasks. Guo et al. proposed Meta‐MGNN, which exploits a molecular graph neural network for learning molecular representations and constructs a meta‐learning framework for model optimization [65]. They further integrated molecular structure, self‐attentive task weights, and attribute‐based self‐supervision modules into the previous framework to leverage unlabeled molecular information. Moreover, Wang et al. noted that different molecular substructures will make the molecules have different properties, and the relationship between molecules will change with the target properties [66]. They proposed a property‐aware relation network (PAR) to take advantage of this fact they found. They introduced a property‐aware embedding module that transforms learned molecular embeddings into a substructure‐aware space associated with target property and designed an adaptive relationship graph learning component to jointly evaluate the molecular relationship graph and complete the molecular embedding with respect to the target property so that limited labels can be spread more efficiently between similar molecules.

## 4 CHALLENGES AND PROSPECTS

Although many deep learning methods have been very successful in molecular property prediction, there are still some unresolved challenges in this field. Here we summarize some of the current critical problems and some perspectives on the future direction of the molecular property prediction field.

First, deep learning methods are data‐driven prediction methods, and the performance of these models directly depends on the quality of the data. However, publicly available high‐quality datasets are still very scarce, and many of them contain only a few thousand or even a few hundred labeled data. Many high‐quality, large‐scale labeled datasets are stored in pharmaceutical companies’ private databases. To address this problem, researchers have proposed a number of methods to leverage unlabeled data, as mentioned earlier. But this approach is a desperate measure, and researchers should work with chemists, pharmacologists, and medical scientists to effectively use accurate data from the wet laboratory.

Second, information about the molecule other than its structure is not effectively used. In the beginning, researchers used only sequence information or 2D topological information of molecules. Subsequently, someone realized the importance of molecular 3D topology and introduced it to molecular property prediction [45]. In addition, some researchers combined molecular 2D topology with 3D geometry in order to learn a more comprehensive representation of molecules [42]. However, researchers have not fully utilized other information about molecules besides their structures, such as surface information and quantum chemical information. We believe that this additional information can further help deep learning models to accurately predict the relevant properties of molecules.

Finally, most of the molecular properties currently predicted using deep learning are not specific to a particular disease, which makes them much less valuable for application. Recently, we are glad to see that many people are aware of this problem and are starting to combine structural information of proteins to predict the affinity of molecules to target proteins [67]. We hope that researchers introduce more bioinformatics knowledge such as transcriptomics into molecular property prediction in future studies in order to directly predict the therapeutic effect of a molecule on a specific disease.

## 5 CONCLUSION

In this review, we summarize several of the most important parts of molecular property prediction models: molecular representations, commonly used datasets, and advanced deep learning methods. Among the advanced deep learning methods, we focus on GNN‐based methods, Transformer‐based methods, 3D pre‐training methods, contrast learning methods, multi‐task learning methods, transfer learning methods, and meta‐learning methods. We present their basic ideas, as well as their practical applications in molecular property prediction. Finally, we point out some urgent problems in the field of molecular property prediction and give our opinion and prospects. We hope that more advanced deep learning methods will be applied to molecular property prediction in the future.

## AUTHOR CONTRIBUTIONS

**Chao Pang:** Conceptualization; visualization; writing – original draft preparation. **Henry H. Y. Tong:** Writing – review and editing. **Leyi Wei:** Project administration; resources; supervision; writing – review and editing.

## CONFLICT OF INTEREST STATEMENT

The authors Chao Pang, Henry H. Y. Tong, and Leyi Wei declare that they have no conflict of interest or financial conflicts to disclose.

## ETHICS STATEMENT

This is a review article and does not involve any research related to human or animal subjects.
